# Randomised Clinical Efficacy Trial of Topiramate and Nitrazepam in Treatment of Infantile Spasms

**Published:** 2014

**Authors:** Razieh FALLAH, Fahimah SALOR, Sedighah AKHAVAN KARBASI, Hadi MOTAGHIPISHEH

**Affiliations:** 1 Department of Pediatrics, Shahid Sadoughi University of Medical Sciences, Yazd, Iran; 2 Growth Disorders of Children Research Center, Shahid Sadoughi University of Medical Sciences, Yazd, Iran; 3 Pediatrician

**Keywords:** Infantile spasms, Topiramate, Nitrazepam, Seizure

## Abstract

**Objective:**

Infantile spasms (IS) are among the most catastrophic epileptic syndromes of infancy. The purpose of this study was to compare efficacy and safety of topiramate (TPM) and nitrazepam (NZP) as first-line drugs in the treatment of IS.

**Materials & Methods:**

In a parallel single-blinded randomized clinical trial, 50 patients with IS referred to Pediatric Neurology Clinic of Shahid Sadoughi University of Medical Sciences, Yazd, Iran, were evaluated from September 2008 to March 2010.

Patients were randomly assigned to two groups to be treated with TPM or with NZP for 6 months. The primary endpoint was efficacy in cessation of all spasms or reduction of more than 50% in weekly seizure frequency, which was evaluated before and 6 months after the drug use. Secondary outcome was clinical sideeffects of the drugs.

**Results:**

Twenty boys (40%) and 30 girls (60%) with the mean age of 9.4±3.8 months were evaluated. Cessation of all spasms occurred in 12 (48%) infants in TPM group and 4(16%) in NZP group. Eight (32%) children in TPM group and 7 (28%) in NZP group had more than 50% reduction in spasms frequency. So, TPM was more effective. Side effects were seen in 32% of TPM and in 36% of NZP groups.

**Conclusion:**

Topiramate is an effective and safe drug, which might be considered as the firstline drug for the treatment of ISs.

## Introduction

Infantile spasms (IS) are among the most catastrophic myoclonic epilepsies of infancy (1). Incidence of IS varies from 0.2 to 0.6 per 100 child, and the peak onset age of the epileptic syndrome is 3 to 7 months. The spasms are bilateral, causing symmetric sudden contractions of neck, trunk, and extremities, and they may be of flexor, extensor or mixed types. Hypsarrhythmia pattern is seen in electroencephalography of such patients, which is composed of a chaotic, bilaterally asynchronous highvoltage polyspike and slow wave discharges interspersed with multifocal spikes and slow waves (2,3).

Etiologic classification of infantile spasms is as follows:

1. Symptomatic: with identifiable prenatal, perinatal, and postnatal causes with developmental delay at the time of presentation

2. Cryptogenic: Unknown underlying cause, normal development at the onset of spasms, normal neurological exam and neuroimaging and no abnormalities in metabolic evaluation.

3. Idiopathic: Pure functional cerebral dysfunction with complete recovery, no residual dysfunction, normal neurodevelopment, normal neuroimaging, and normal etiologic evaluation. This class is based on final outcome and cannot be certainly confirmed according to the history and presenting symptoms. The terms idiopathic and cryptogenic have been synonymously used in many studies (2,4-6).

Since early control of spasms may improve prognosis and is accompanied by a higher chance for normal developmental outcome, treatment should be begun immediately (5-7).

There is no agreement on the first choice of the drug for treatment of IS and no single treatment regimen could be considered superior to others. ACTH or oral corticosteroids, pyridoxine, vigabatrin, and other drugs such as valproic acid, nitrazepam (NZP), topiramate (TPM), zonisamide, lamotrigine, levetiracetam, felbamate, ganaxolone, liposteroid, thyrotropinreleasing hormone, intravenous immunoglobulin, and also ketogenic diet might be used in treatment of spasms as first or second line drugs (2,7). 

ACTH is expensive and hard to obtain in Iran and it has many side-effects, including hypertension, infection, electrolyte abnormalities, hyperglycemia, weight changes, brain shrinkage, sleep and behavioral abnormalities, subdural hematoma/effusion, development of new seizure types, and even death (2,7). Irreversible visual field defect was seen in 30 to 50% of children who were treated with vigabatrin (8). 

It is difficult to recommend for treatment of IS in the absence of comparative trials. So, new antiepileptic drugs with greater efficacy and fewer side-effects are fiercely needed. TPM is one such an agent, and it may also be used as a second-line agent in treatment of IS of tuberous sclerosis, and as a first-line agent for other symptomatic IS treatments. Its anticonvulsant effect is based on the following mechanisms: 

a) Blocking of voltage-dependent Na channels; 

b) Enhancing the inhibitory activity of GABA;

c) Inhibition of excitatory neurotransmission by blocking Kainate /AMP glutamate receptors;

d) Inhibition of erythrocyte carbonic anhydrase (2,7, 9-16).

There is no consensus on mean daily dose of TPM (10), and up to 35 mg/kg/day has been used in IS (7,10,11,16) but maximum dose of 12 mg/kg/day is more effective and safer (13,14,17).

The purpose of this study was to compare clinical efficacy and safety of TPM and NZP as first-line drugs in the control of infantile spasms in Yazd, Iran.

## Materials & Methods

A randomized single–blind clinical, open-label, parallel group study was conducted on patients with ISs who were referred to Pediatric Neurology Clinic of Shahid Sadoughi University of Medical Sciences, Yazd, Iran, from September 2008 to March 2010.

Eligible participants included two months to two years old children who had ISs, and did not use any antiepileptic drugs, ACTH and/ or oral corticosteroids. 

Exclusion criteria consisted of presence of metabolic acidosis, kidney dysfunction, renal stone, and those who had not completed 6 months of treatment period.

The diagnostic criteria for the IS in this study were based on International League against Epilepsy (ILAE), which were classified and done with two groups: symptomatic and cryptogenic (4). 

The developmental status of the patients was assessed by a pediatric neurologist according to Denver II Developmental Screening test. 

Equal randomization was used in this trial, and the allocation ratio was 1:1 for the two groups.

Simple randomization was done using a computer generated random number list, which was prepared by an investigator with no clinical involvement in the trial, and no restriction was exerted. First of all, a research resident obtained parents’ consent and called a person who was independent of the recruitment process for allocation consignment.

The trial adhered to established procedures to maintain separation between the persons who took outcome assessment and the staff who delivered the intervention. The drug which was packaged and labeled according to a medication code schedule generated before the trial, was delivered by the nurses of the clinic. Inside of all packages, amount of the drug in one tablet and the dosage were written. After opening the packages, drug dosage was determined by pediatric neurologist based on each child’s weight.

Primary and secondary outcomes were assessed by a research resident who was not informed of the drug group assignment. Investigators, staff, and participants were all kept masked to outcome measurements and trial results.

Informed consent was taken from the patients’ parents, and the study was approved by the Ethics Committee of Shahid Sadoughi University of Medical Sciences, Yazd, Iran.

Sample size based on Z formula and confidence interval of 95% with 80% power, type one error of 5%, good response (more than 50% decrease in weekly seizure frequency during the follow-up period) of 85% for TPM treatment in another study (18), and an effect size (difference in frequency of good response between the two groups) of 40% for the primary efficacy end point, was assessed in 22 patients per group. For more accuracy, total sample size was determined to be 50 children.

After evaluation for inclusion and exclusion criteria, eligible patients were distributed into two groups. In group one, 25 infants were treated with TPM and in group two, 25 patients were treated with NZP.

In both groups, the drugs were administered orally in two divided doses and were started with lowest dosage to minimize side effects and then, dosage was increased to maximum in a weekly period or to a dosage which controlled seizures in a four-week period as follows: 3, 6, 9, 12 mg/kg/day in patients who took TPM and 0.5, 0.7, 0.9, 1 mg/kg/day in those for whom NZP was used.

During the treatment period, patients were followed up by a pediatric resident of research and in monthly consecutive visits of the patients in the Pediatric Neurology Clinic of Shahid Sadoughi University of Medical Sciences, Yazd, Iran, clinical information about seizure frequency and the drugs’ side-effects were obtained by interview with the patients’ parents, and information of physical examination data which were recorded on questionnaires.

At the end of the 6-month follow up period, drug efficacy and safety were evaluated. Weekly seizure frequency was compared with its related number, before and 6 months after the drug use, and the following classification was done:

1. Seizure-free: all the spasms stopped 

2. Improved: more than 50% reduction in spasms frequency

3. Unchanged: no notable changes was seen in spasms frequency

4. Worsened: spasms frequency increased more than 25%

Cessation of all spasms or more than 50% of reduction in weekly seizure frequency was considered as effective and good response.

Video–EEG monitoring facilities were not available in our city and cessation of clinical seizures was indicative of successful management of IS.

Primary endpoint was efficacy in cessation of all seizures or reduction of more than 50% in weekly seizure frequency which was evaluated before and 6 months after drug use. 

Secondary outcome was clinical side-effects of the drugs in the duration of treatment. The data were analyzed using SPSS statistical software (version 15). Chi-square test or Fisher’s exact test were used for data analysis of qualitative variables, and mean values were compared using independent t-test. 

Differences were considered significant at p-values of less than 0.05. An iInformed consent was taken from the patients’ parents. The study was approved by the Ethics Committee of Shahid Sadoughi University of Medical Sciences, Yazd, Iran.

This study is registered in Iranian clinical trials with registration number IRCT138808052639N1. The researchers got no support from the drugs company. The design and conduct of this trial were straightforward, and we did not have any losses to follow-up or exclusions.

## Results

Twenty boys (40%) and 30 girls (60%) with the mean age of 9.4±3.8 months (range=3-20 months) were evaluated. Symptomatic IS was seen in 43 (86%) patients. Onset age of seizures was 4.92±3.2 months in symptomatic IS and 7.52±2.4 months in cryptogenic IS (p=0.04).

Forty-three children (86%) had neurodevelopmental delay, and family history of epilepsy was positive in 14% (N=7) of them. EEG showed hypsarrhythmia in 40 (80%) children.

Neuroimaging results were normal in 18 (36%) children, and abnormal results were brain atrophy in 20 (40%), intracranial calcification in 6 (12%), structural CNS dysgenesia in 5 (10%), and hydrocephaly in one of patients (2%).

From viewpoint of spasms types, 18 patients (36%) had mixed, 17 (34%) had flexor, and 15 showed extensor (30%). Comparison of some clinical and paraclinical characteristics of patients are shown in Table 1, which indicates that sex distribution, type of IS, positive family history of epilepsy, etiologic class of IS, EEG and neuroimaging results, mean of age, age of seizure onset, and number of spasms clusters per week were not statistically significantly different between the two groups. 

Results of efficacy analysis are illustrated in Table 2, which indicate that TPM was more effective in the control of spasms. 

After 6 months of treatment, good response (stopping of all seizures or more than 50% reduction in weekly seizure frequency) was seen in 20 infants (80%) of TPM group (95% confidence interval: 0.64-0.96), and in 11 patients (44%) of NZP group (95% confidence interval: 0.24-0.63). Therefore, TPM was significantly more effective (p=0.009).

Frequency distribution of good response based on etiologic class of IS and EEG and neuroimaging results is shown in Table 3, which indicates that no statistically significant differences were seen from these viewpoints.

Mean dose of TPM for seizure control was 6.9±3.4 mg/ kg/day, and it was 0.7±0.3 mg/kg/day for NZP. 

No serious paraclinical adverse events, such as hematologic abnormality, hepatotoxicity, and nephrotoxicity were seen in the two groups.

Clinical side effects were seen in 32% (N=8) of the patients in the TPM group (lethargy in three, hypotonia in two, hyperthermia in two, anorexia and weight loss in one), and in 36% (N=9) of NZP group (sialorrhea in four, and lethargy in three, hypotonia in one and anorexia and weight loss in one). No statistically significant differences were seen from viewpoint of safety between the two drugs (p=0.76).

All side effects disappeared in one or two weeks and treatment was stopped in none of patients who suffered from them.

## Discussion

In this study, 86% of patients had symptomatic IS among whom inborn error of metabolism was the most common cause.

In a study Thailand, 45.8% of patients had symptomatic and the most common etiology in symptomatic cases was hypoxic ischemic encephalopathy (19).

In karvelas et al study, 63% had symptomatic IS, and cortical dysgenesia was the most frequent cause (20). 

In a study in Taiwan, 80% of patients had symptomatic IS, and tuberous sclerosis, asphyxia, CNS malformation were the most common causes (21) and in another study, 70% were symptomatic and brain malformations and tuberous sclerosis were seen in 35% of patients (22).

In Zagreb, 81.2% had symptomatic IS, and hypoxicischemic encephalopathy was the most common etiologic factor (2) and 84% were symptomatic in Peltzer et al.’s study (11).

In the present study, 80% of patients had hypsarrhythmia pattern in EEG. Based on pediatric neurology textbook, hypsarrhythmia may be seen in 66% of patients and it is most obvious in non-rapid eye movement (non- REM) sleep (3). This EEG pattern appears after 3-4 months of age and may only be present periodically and may change or even be resolved by the passage of time. Moreover, during REM sleep and immediately after arousal from REM or non-REM sleep, EEG can be normal for up to several minutes (2,3). 

In this study, good response to drugs was not different in symptomatic and cryptogenic IS, which is not in accordance with other studies (6,7,11).

In the present study, control of all spasms by TPM treatment was seen in 48% of patients. However, in other studies, this rate varied between 16.7% and 57.4% (10-16,20).

In the present study, more than 50% of reduction in seizure frequency was seen in 32% of children with TPM treatment, But the rate had been 33% (15), 36% (16), 47% (13), and 85% (18) in other studies. 

Possible explanations for these discrepancies are differences in sample size, duration of treatment, selection method of patients, and dosage of the drug. 

Dose of TPM was 3-12 mg/kg/day in this study, while in other studies 25 mg/kg/day (16), 3.57-20 mg/kg/ day(12), 1-12 mg/kg/day (14), 3-27 mg/kg/day (15), and 1-10 mg/kg/day (24) of the drug had been used. 

In our study, the mean dose of TPM for seizure control was 6.9±3.4 mg/kg/day, which is almost similar to a Taiwanese study on 13 children, in whom 7.35±4.9 mg/kg/day of the drug was used. (18). 

In this study, TPM adverse effects were seen in 32% of children, and lethargy was the most common sideeffect. 

TPM may induce metabolic acidosis, especially in patients with renal diseases or in those who are on a ketogenic diet or zonisamide (9).

In Mikaeloff et al.’s study, the most common adverse effects were neurobehavioral problems (drowsiness, fatigue, and hyperactivity) and gastrointestinal disorders (anorexia and loss of appetite) (10).

In Pletzer et al. study, 10.5% of patients (2 out of 19) showed side-effects, such as appetite loss, tremors, and lethargy (11).

In Zou et al. studies in china, 38.8% had adverse effects, most common of which were anorexia and somnolence (12).

In Korinthenberg et al.’s study in Germany, adverse effects were seen in 25% of patients, and sedation, loss of appetite, weight loss, and metabolic acidosis were the most common side-effects (13).

In Hosain et al.’s study, irritability was the most common side-effect (15).

In a study by Grosso et al., adverse effects, including weight loss, hyperthermia, sedation, and nervousness were seen in 58% of children, and the majority of the side-effects then disappeared after slow titration or decrease of drug dose (25).

In this study, control of all spasms and more than 50% of reduction in seizure frequency with NZP treatment was seen in 16% and 28% of patients, respectively, and adverse effects were seen in 36% of children, of which sialorrhea was the most common side-effect. 

In a study in Thailand, effectiveness of sodium valproate with or without NZP or clonazepam was evaluated in treatment of IS, and they concluded that sodium valproate should be used concomitantly with benzodiazepines, especially clonazepam (19). 

In Capovilla et al.’s study, three cases with West syndrome were treated with NZP in dose of 0.7-1.5 mg/kg/day. They were evaluated 6 months after the discontinuation of the drug, and no cases had recurrence of spasms (26).

In an open label study, 20 children with medically refractory infantile spasms or Lennox-Gastaut syndrome were treated with NZP in dose of 0.5-3.5 mg/kg/day. Control of all seizures and more than 50% reduction in seizure frequency were respectively seen in 25% and 35% of them, and side-effects were oral hypersecretion in 60% and sedation in 30% (27).

In a study on evaluation of the effectiveness of NZP in Lennox-Gastaut Syndrome, the most common sideeffects were drooling in 60% and sedation in 40% of their patients (28).

Sudden death and higher mortality were reported in epileptic children who received NZP (29), and tolerance to drug and significant adverse effects of NZP has deterred its more use (30).

In conclusion, training of parents and children health care providers for early reference, diagnosis, and treatment of ISs is necessary. TPM is an effective and safe drug in treatment of ISs, which should be considered as first line of treatment.

**Table1:** Comparison of Clinical and Paraclinical Characteristics of Patients In Both Groups

Data			Topiramate	Nitrazepam	P.Value
Sex	Female		13	17	0.248
	Male		12	8	
Type of infantile spasms	Flexor		8	9	0.46
	Extensor		6	9	
	Mixed		11	18	
Family history of epilepsy	Yes		1	6	0.14
	No		24	19	
Hypsarrhythmia in EEG	Yes		20	20	1
	No		5	5	
Neuroimaging results	Abnormal		15	17	0.55
	Normal		10	8	
Etiologic class	Symptomatic	Tuberous sclerosis	1	2	0.22
		Inborn error of metabolism	5	6	
		Chromosomal & Genetic syndrome	0	3	
		CNS dysgenesia	5	3	
		Congenital infections	3	2	
		Birth asphyxia	4	3	
		Others	5	1	
	Cryptogenic		2	5	
Age in months (mean ± SD)			9.01 ± 3.96	9.82 ± 3.76	0.491
Age of seizure onset in months (mean ± SD)			5.39 ± 2.97	5.22 ± 3.55	0.854
Clusters number in a week (mean ± SD)			35.16 ± 28.27	26.16 ± 20.89	0.238

**Table 2:** Efficacy Results of The Two Drugs After Six Months of Treatment

**Drug**	**Topiramate**		**Nitrazepam**		**Total**		**P.Value**
**Response**	**Number**	**Percent**	**Number**	**Percent**	**Number**	**Percent**	
Seizure free	12	48	4	16	16	32	0.03
Improved	8	32	7	28	15	30	
Unchanged	4	16	13	52	17	34	
Worsened	1	4	1	4	2	4	
Total	25	100	25	100	50	100	

**Table 3:** Frequency Distribution of Good Response Based On Etiologic Class of Infantile Spasms, Electroencephalography and Neuroimaging Results

**Good response**		**Yes**		**No**		**P.Value**
**Data**		**Number**	**Percent**	**Number**	**Percent**	
Etiologic class	Symptomatic	28	65	15	35	0.26
	Cryptogenic	3	43	4	57	
Neuroimaging results	Abnormal	19	59	13	41	0.6
	Normal	12	67	6	33	
Hypsarrhythmia in EEG	Yes	24	60	16	40	0.56
	No	7	70	3	30	
